# Fusion of *EML4 *and *ALK *is associated with development of lung adenocarcinomas lacking *EGFR *and *KRAS *mutations and is correlated with ALK expression

**DOI:** 10.1186/1476-4598-9-188

**Published:** 2010-07-13

**Authors:** Xuchao Zhang, Shirley Zhang, Xuening Yang, Jinji Yang, Qing Zhou, Lucy Yin, Shejuan An, Jiaying Lin, Shiliang Chen, Zhi Xie, Mike Zhu, Xiaolin Zhang, Yi-long Wu

**Affiliations:** 1 Medical Research Center of Guangdong General Hospital, Guangdong Lung Cancer Institute, Guangdong Academy of Medical Sciences, Guangzhou 510080, China; 2 Guangdong General Hospital-AstraZeneca Innovation Center China Joint Laboratory, Guangzhou 510080, China

## Abstract

**Background:**

The anaplastic lymphoma kinase (*ALK*) gene is frequently involved in translocations that lead to gene fusions in a variety of human malignancies, including lymphoma and lung cancer. Fusion partners of *ALK *include *NPM*, *EML4*, *TPM3*, *ATIC*, *TFG*, *CARS*, and *CLTC*. Characterization of ALK fusion patterns and their resulting clinicopathological profiles could be of great benefit in better understanding the biology of lung cancer.

**Results:**

RACE-coupled PCR sequencing was used to assess *ALK *fusions in a cohort of 103 non-small cell lung carcinoma (NSCLC) patients. Within this cohort, the *EML4*-*ALK *fusion gene was identified in 12 tumors (11.6%). Further analysis revealed that *EML4*-*ALK *was present at a frequency of 16.13% (10/62) in patients with adenocarcinomas, 19.23% (10/52) in never-smokers, and 42.80% (9/21) in patients with adenocarcinomas lacking *EGFR *and *KRAS *mutations. The *EML4*-*ALK *fusion was associated with non-smokers (*P *= 0.03), younger age of onset (*P *= 0.03), and adenocarcinomas without *EGFR*/*KRAS *mutations (*P *= 0.04). A trend towards improved survival was observed for patients with the *EML4*-*ALK *fusion, although it was not statistically significant (*P *= 0.20). Concurrent deletion in *EGFR *exon 19 and fusion of *EML4*-*ALK *was identified for the first time in a Chinese female patient with an adenocarcinoma. Analysis of ALK expression revealed that ALK mRNA levels were higher in tumors positive for the *EML*-*ALK *fusion than in negative tumors (normalized intensity of 21.99 vs. 0.45, respectively; *P *= 0.0018). However, expression of EML4 did not differ between the groups.

**Conclusions:**

The *EML4*-*ALK *fusion gene was present at a high frequency in Chinese NSCLC patients, particularly in those with adenocarcinomas lacking *EGFR*/*KRAS *mutations. The *EML4*-*ALK *fusion appears to be tightly associated with ALK mRNA expression levels. RACE-coupled PCR sequencing is a highly sensitive method that could be used clinically for the identification of *EML4*-*ALK*-positive patients.

## Background

The anaplastic lymphoma kinase (*ALK*) gene encodes a receptor tyrosine kinase (RTK) that has been discovered to be present in a number of fusion proteins consisting of the intracellular kinase domain of *ALK *and the amino-terminal portions of different genes [1,2]. Activated ALK is involved in the inhibition of apoptosis and the promotion of cellular proliferation through activation of downstream PI3K/Akt and MAPK signalling pathways [3]. Genetic alterations involving ALK, including gene fusions, amplification, and mutations, have been identified in anaplastic large cell lymphomas, inflammatory myofibroblastic tumors, and neuroblastoma, respectively [1,4-7]. To date, in studies from a variety of human cancer types, the reported fusion partners of *ALK *have included *NPM *[1], *EML4 *[8], *MSN *[9], *TPM3 *[10,11], *ATIC *[12-14], *TFG *[15], *CARS *[16], *CLTC *[17], and *KIF5B *[18]. In lung cancer, the primary *ALK *fusion detected was identified as *EML4*-*ALK*, followed by *TFG*-*ALK *and *KIF5*-*ALK*, although other unknown fusions may also exist which can not be detected due to limits of present technology [19].

The *ALK*-*EML4 *fusion attaches the *ALK *gene to a gene involved in microtubule formation and stabilization, "echinoderm microtubule associated protein-like 4" (*EML4*) [20,21]. This fusion generates a transforming fusion tyrosine kinase, several isoforms of which have been identified in lung cancers [8,22]. The frequency of the *EML4*-*ALK *fusion was first reported by Soda *et al*. to be approximately 6.7% (5/75) in non-small cell lung carcinomas (NSCLCs) in Japanese patients [8]. *ALK *and *EML4 *are both located on the short arm of chromosome 2, separated by 12 megabases (Mb) of sequence, and are oriented in opposite directions. To date, more than nine different variants of the *EML4*-*ALK *fusion have been identified. These variants consist of exons 20 to 29 of *ALK *fused to *EML4 *exon 13 (variant 1, V1), exon 20 (V2), exon 6 (V3a), exon 6 with an 11 amino acid (aa) insertion (V3b), exon 14 with an additional 11 nucleotide insertion of unknown origin at nucleotide 50 in exon 20 of *ALK *(V4), exon 2 (V5), exon 13 (V6), exon 14 with the fusion beginning at nucleotide 13 in exon 20 of *ALK *(V7), exon 15 (primarily also reported as "V4", in this article depicted as V8), and exon 18 ("V5", accordingly here as V9), as described in detail in Horn and Pao's review [23]. *ALK *gene fusions have been demonstrated to be oncogenic in 3T3 and Ba/F3 cellular models [24]. Although different variants of EML4-ALK fusion proteins may exhibit different enzymatic activities, EML4 retains the N-terminal coil-coiled domain (CC) in all EML4-ALK variants. This domain has been shown to be responsible for the dimerization and constitutive activation of EML4-ALK [24]. Importantly, in some cell lines harboring *EML4*-*ALK *fusions, targeting of ALK using specific inhibitors has shown promising efficacy for treatment of lung cancer through inhibition of Akt and induction of apoptosis. For this reason, ALK inhibitors have been developed and assessed in early clinical trials [25,26].

Published studies using reverse transcriptase-polymerase chain reaction (RT-PCR) analysis or fluorescent *in situ *hybridization (FISH) to characterize *EML4*-*ALK *fusions in lung cancer have revealed frequencies ranging from 0.5% to 7.5% in general lung cancer patients and a frequency of 13.5% in clinical factor-enriched cases [23], [27,28]. Here, we report the development of a technology based on rapid amplification of cDNA ends (RACE)-coupled PCR and sequencing to analyze expression of *ALK *fusion genes. This technology was designed to identify both known and novel fusion partners of *ALK*, followed by confirmation using qualitative specific RT-PCR. Using this method, we analysed the *ALK *fusion status in clinical lung cancer samples. Results from this study could, thus, provide a better understanding of *ALK *fusions with all potential partner genes in a clinical population. Furthermore, these results provide insight into the clinicopathological characteristics of *ALK *fusion-positive Chinese NSCLC patients.

## Results

### Identification of *EML4*-*ALK *fusions in 103 cases of NSCLC

**Figure 1:**
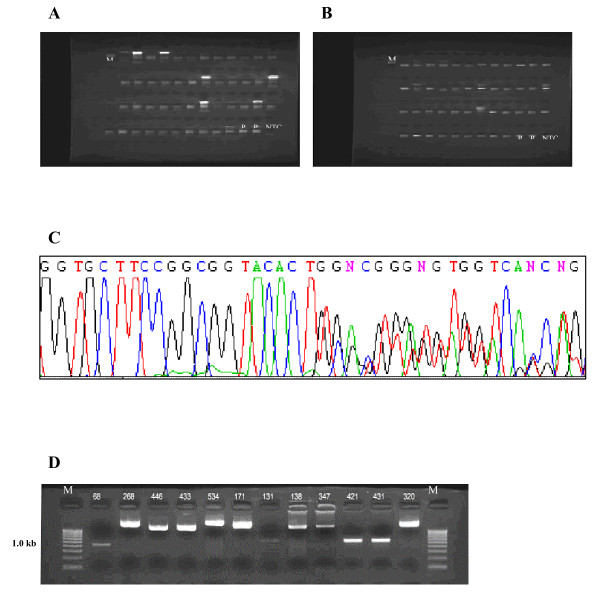
**Assessment of *ALK *fusion status by RACE-coupled PCR sequencing**. (A) Representative image showing gel electrophoresis results for first-round PCR products. Lane M, marker (200 bp ladder); lanes P, positive controls (2 lanes); lane NTC, no template control; other lanes, samples 1 through 45. (B) Representative image showing gel electrophoresis results for second-round PCR products. Lane organization is identical to (A). (C) Representative sequencing chromatograph showing *ALK *fusion with *EML4*. (D) RT-PCR validation for samples positive for *ALK *fusion expression. RT-PCR was used to confirm *EML4*-*ALK *fusion transcript expression in 12 samples. Sample 68 is the V5 variant (E2;A20, 408 bp). Samples 268, 171, and 320 are V9 variants (E18;A20, 2256 bp). Samples 446, 433, 138, and 347 are V1 variants (E13;A20, 1689 bp). Sample 534 is the V2 variant (E20;A20, 2445 bp). Samples 131, 421, and 431 are V3a/b variants (E6;A20 or E6 ins33;A20, 867 and 900 bp, respectively). Lane M, marker (100 bp ladder).

RNA samples from a total of 103 NSCLC cases were reverse-transcribed to make cDNA, followed by oligo-dC tailing. Two successive rounds of PCR were used to identify potential fusion fragments (Fig. 1A, B). BigDye3.1-labeled products were then sequenced to identify fusions between *ALK *and potential partners (Fig. 1C). Based on a sequence alignment with the *ALK *reference sequence (NCBI accession number: NM_004304.3), in total, 12 samples were identified as *ALK *fusion-positive (Table 1).

**Table 1:** *EML4*-*ALK *fusion variants identified in the 12 NSCLC cases.

Variant	Published fusion type*	No. of positive samples	Frequency
V1	EML4 E13-ALK E20	4	3.9%
V2	EML4 E20-ALK E20	1	1.0%
V3a	EML4 E6-ALK E20	3	2.9%
V3b	EML4 E6-11 aa in INS6-ALK E20		
V4	EML4 E15-ALK E20	0	0
V5	EML4 E2-ALK E20	1	1.0%
V6	EML4 E18-ALK E20	3	2.9%

*All fusion types have previously been published.

### RT-PCR confirmation of expression of ALK fusion transcripts in positive samples

Fusion gene-specific primers were designed, and qualitative RT-PCR was conducted to confirm the presence of *ALK *fusions in positive samples identified by RACE-coupled PCR sequencing. Fusion RNAs from the 12 *ALK *fusion-positive samples were amplified by RT-PCR, and specific bands corresponding to the expected products were observed after gel electrophoresis (Fig. 1D).

### Correlation of *ALK *fusion with ALK expression

**Figure 2:**
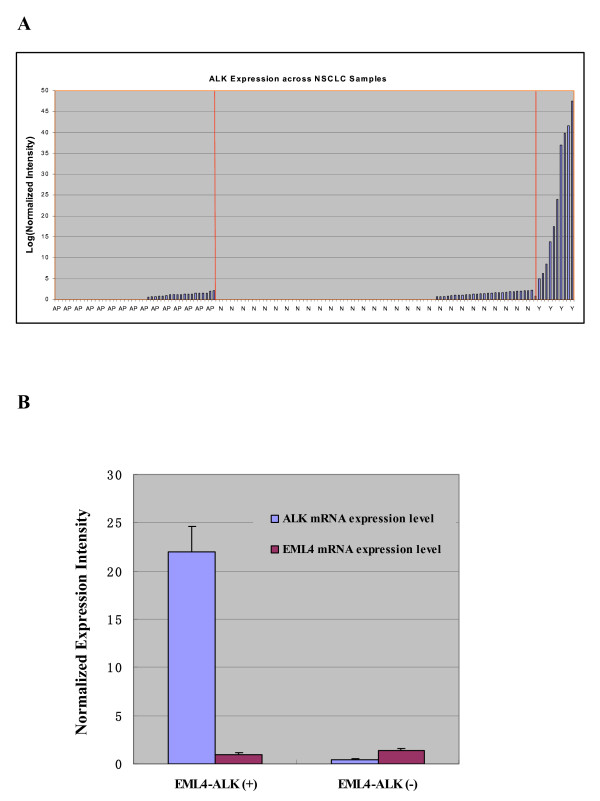
**ALK expression is associated with *ALK *fusion status**. (A) ALK is overexpressed at the mRNA level in 10 fusion-positive cases analyzed using Affymetrix Genechip^® ^U133 plus 2.0. AP, Adjacent normal tissues; N, Cancer samples with no fusion; Y, Cancer samples positive for fusion. (B) ALK is differentially expressed between *EML*-*ALK *fusion-positive and negative samples (normalized intensity of 21.99 vs. 0.45, respectively; *P *= 0.0018), but EML4 expression was not significantly different between the groups. Error bars indicate mean ± standard deviation.

Gene expression profiling was conducted on clinical samples using the Affymetrix GeneChip^® ^Human Genome U133 plus 2.0 technology. Normalized expression intensities for the ALK and EML4 transcripts were extracted and plotted versus *ALK *fusion status (Fig. 2A). ALK was found to be significantly over-expressed in the 10 fusion-positive samples analyzed (2 *EML4*-*ALK *cases were not analyzed by microarray), but not in samples lacking *ALK *fusions or control adjacent tissues. ALK expression was significantly different in *EML*-*ALK *fusion-positive and -negative samples (normalized intensity values of 21.99 vs. 0.45, respectively; *P *= 0.0018). In contrast, EML4 expression did not differ significantly between the groups (Fig. 2B). These results indicate that presence of the *ALK *fusion was strongly associated with ALK mRNA expression levels, leading to a 50-fold increase in expression.

### Concurrent *EGFR *mutation and *ALK *fusion in one sample

**Figure 3:**
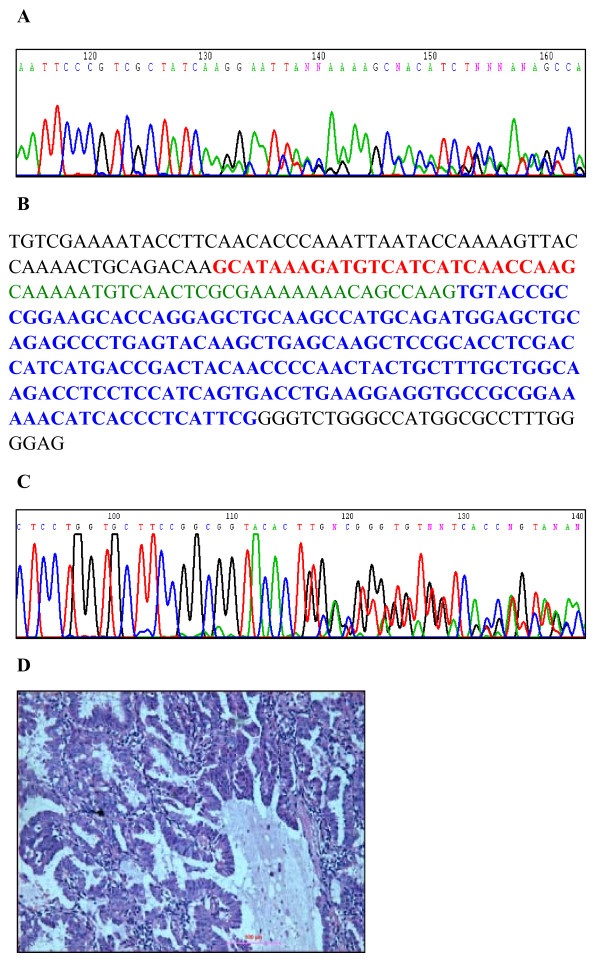
**Concurrent heterozygous *ALK *fusion and *EGFR *deletion in a single adenocarcinoma**. (A) Chromatograph indicating a heterozygous deletion (2235-2249 del 15) in exon 19 of *EGFR *in a patient with *EML4*-*ALK*. (B) Sequence of *EML4*-*ALK *fusion variant 3b. Red sequence indicates exon 6 of *EML4*. Green sequence indicates an insertion of 33 bp from intron 6 of *EML4 *found in variant 3b. Blue sequence indicates exon 20 of *ALK*. (C) Chromatograph for *EML4*-*ALK *fusion variant 3b indicating the presence of a heterozygous fusion of *ALK *with exon 6 of *EML4 *with an additional 33 bp insertion. (D) Histological staining of the adenocarcinoma from the patient with both an *EGFR *mutation and *EML4*-*ALK *fusion. Scale bar = 100 μm.

For all samples analyzed for *ALK *fusion, DNA was obtained for sequencing of the *EGFR *gene to assess mutation status. In one sample, the patient was found to be heterozygous for 2235-2249 del15 in exon 19 of *EGFR *(Fig. 3A). In the same sample, *EML4*-*ALK *variant 3b was also present, in which exons 20 to 29 of *ALK *were connected to exon 6 of *EML4*, with an additional 33 bp insertion from intron 6 of *EML4 *(Fig. 3B, C). Histological adenocarcinoma was identified in this patient by morphological examination of hematoxylin and eosin-stained tissue samples (Fig. 3D).

### Association of *ALK *fusion status with clinicopathological parameters

Of the 12 samples containing *EML4*-*ALK *fusions, 10 were identified as adenocarcinomas (one of which contained a mixed squamous carcinoma component), and two were identified as squamous cell carcinomas. In these patients, the presence of the *ALK *fusion was mutually exclusive with the presence of *KRAS *mutations. Notably, although the presence of *ALK *fusions was correlated with wild-type *EGFR *status (*P *= 0.04), we did identify one patient who had both the *EML4*-*ALK *fusion and an *EGFR *mutation.

**Figure 4:**
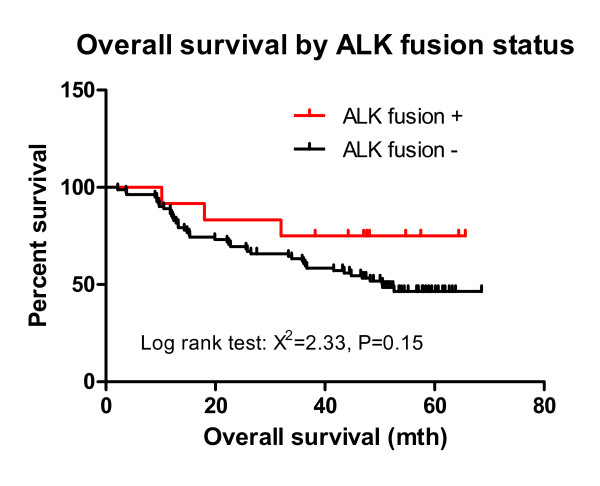
**Kaplan-Meier survival curve, stratified by *ALK *fusion status**. Percent survival of patients with and without *ALK *fusions was plotted versus the number of months after surgery. Prior to the date of article submission, the *ALK *fusion-positive patients (*n *= 12) did not have enough events to determine the median overall survival, while the *ALK *fusion-negative patients (*n *= 101) had a median overall survival of 50.5 months. The hazard ratio for death in the *ALK *fusion-positive group was 0.54 (95% CI, 0.23-1.26). The log-rank (Mantel-Cox) test had a chi-squared value of 2.33 (*P *= 0.15).

To our knowledge, this is the first patient identified with a concurrent *EGFR *exon 19 deletion and the *EML4*-*ALK *fusion translocation. The concurrent mutations occurred in a female, non-smoking Chinese patient with a histological adenocarcinoma. *EML4*-*ALK *was significantly associated with non-smoking (*P *= 0.03). Patients with *ALK *fusions exhibited a significantly decreased number of smoking pack-years (5.0 vs. 18.5; *P *< 0.01) and were younger (53 vs. 61; *P *= 0.03) compared with patients without the fusion gene. No mutation in the kinase domain of *ALK *was detected by sequencing samples from the first group of 50 NSCLC cases collected consecutively (data not shown). Thus, mutation of *ALK *in NSCLC likely either does not occur or is rare. A trend towards improved survival was observed in the *EML4*-*ALK *cohort, though this was not statistically significant (*P *= 0.15; Fig. 4). *ALK *fusion-positive NSCLCs exhibited a hazard ratio of 0.54 (95% CI, 0.23-1.26) for overall survival.

## Discussion

**Figure 5:**
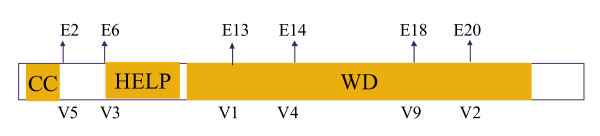
**Structure of the N-terminal exons of *EML4***. The coiled-coil domain (CC) is responsible for dimerization and constitutive activation of *EML4*-*ALK*; HELP, hydrophobic EMAP-like protein domain; WD, WD-repeat domain.

*ALK *belongs to the insulin receptor subfamily of receptor tyrosine kinases. Aberrant ALK activity has recently been shown to be present in anaplastic large cell lymphoma, as well as in solid tumors, including NSCLC [8,22]. Previous investigations have shown that translocation of *ALK *can result in fusion with the neighbouring gene, *EML4*, in cancer cells [19]. The fused genes then encode a fusion protein in which the intracellular tyrosine kinase domain of the ALK receptor is constitutively active. In all *EML4*-*ALK *fusion variants, the amino-terminal coiled-coil (CC) domain of EML4 has been shown to be retained in the fusion protein and is believed to be responsible for receptor dimerization and constitutive activation of the kinase domain (Fig. 5).

In the present study, the five transcript variants identified all possessed the CC domain and are, thus, likely to produce active, oncogenic EML4-ALK proteins in these NSCLC tumors. In future studies, we intend to investigate ALK kinase activity and activation of downstream signalling kinases in patient samples. These studies are particularly important, as data from Soda *et al*. [8] revealed that deletion of either the HELP or WD domains of EML4 can reduce kinase activity, by up to 50%. Thus, some variants may produce kinases with more activity than others.

To determine whether *ALK *fusions exhibit characteristic expression profiles at the mRNA level, we compared *ALK *expression based on microarray data (using the Affymetrix Genechip^® ^human genome U133 plus 2.0 system) that corresponded to the same set of RNA samples. We found that ALK was expressed at higher levels in samples containing *EML4*-*ALK*, compared with samples that did not contain the fusion. Due to potential errors in microarray expression data arising from multiple correction procedures, real-time qRT-PCR should be performed to confirm ALK mRNA expression data. Additionally, use of immunohistochemistry could also confirm whether mRNA expression is correlated with EML4-ALK protein expression.

Careful review of the literature reveals that ALK fusion proteins are frequently present in lung cancer patients (Table 2). For example, Soda *et al*. found that the frequency of *EML4*-*ALK *fusion variants 1 and 2 was 6.7% (5/75) in a Japanese population using RT-PCR [20]. Taheuchi *et al*. [29] found a frequency of 4.35% (11/253) for *EML4*-*ALK *fusion variants 1-5 in Japanese patients using RT-PCR. Another RT-PCR-based study, by Koivunen *et al*. [30], showed that 3.6% (6/167) of Korean patients and 1.4% (2/136) of Caucasian patients with lung cancer possessed *EML4*-*ALK *variants 1, 3, and 4. Using a combination of FISH and RT-PCR, Perner *et al*. [31] found that 0.5% (3/603) of Caucasians with lung cancer have *EML4*-*ALK *variant 1. Wong *et al*. [32] found that *EML4*-*ALK *variants 1, 2, 3, and 9 (previously as "V5") were present at a frequency of 4.9% (13/266) in Chinese patients with lung cancer using RT-PCR. Also using RT-PCR, Martelli [33] found that 7.5% (9/120) of Italian and Spanish patients possessed *EML4*-*ALK *variants 1 and 3. In a study by Shaw and colleagues, NSCLC patients were selected for genetic screening on the basis of two or more of the following characteristics: female gender, Asian ethnicity, never/light smoking history, and adenocarcinoma histology. Analysis of *EML4*-*ALK *in these patients by FISH revealed a substantially higher frequency of the fusion (13%; 19/141) [34]. As is evident in these studies, RT-PCR and FISH represent the primary methods previously used to analyze the frequency of *ALK *fusions (Table 2).

**Table 2:** ALK fusion variants previously reported in lung cancer.

Citation	**Freq**.	Origin	Method	Number of Positives for each Fusion type								
				V1	V2	V3	V4	V5	V6	V7	V8	V9
*Soda, et al. Nature 2007*	6.7% (5/75)	Japanese	RT-PCR	**3**	**2**							
*Takeuchi et al*. *Clin Cancer Res. 2008 *	4.35% (11/253)	Japanese	RT-PCR	**3**	**3**	**3**	**1**	**1**				
*Takeuchi et al*. *Clin Cancer Res. 2009 *	/	Japanese	iAEP/RT-PCR	**1**	**1**	**3**			**1**	**1**		
*Koivunen et al*. *Clin Cancer Res. 2008 *	3.6% (6/167)	Korean	RT-PCR	**2**		**4**					**2**	
	1.4% (2/136)	Caucasian										
*Inamura et al. J Thorac Oncol. 2008*	2.3% (5/221)	Japanese	RT-PCR	**2**	**3**							
*Perner et al. Neoplasia 2008*	0.5% (3/603)	Caucasian	FISH, RT-PCR	**3**								
*Wong et al*. *Cancer. 2009 *	4.9% (13/266)	Chinese	RT-PCR	**2**	**2**	**8**						**1**
*Martelli et al*. *Am J Pathol. 2009 *	7.5% (9/120)	Italian and Spanish	RT-PCR	**7**		**2**						
***Present study***	**11.6%** **(12/103)**	**Chinese**	**RACE-coupled PCR sequenc-ing**	**4**	**1**	**3**		**1**				**3**

Our data, which was based on non-selective hospital-obtained samples, revealed a frequency of 11.6% (12/103) for *EML4*-*ALK *fusion variants 1, 2, 3, 5, and 9 in Chinese NSCLC patients using RACE-coupled PCR sequencing technology. This rather high frequency of *ALK *fusion may be attributable the high sensitivity of RACE-PCR sequencing assays. Use of this technique has also confirmed that, in non-small cell lung cancer, the fusion of *ALK *appears to occur solely with *EML4*, as previously reported [35], [36], rather than with other genes.

Comparison of *ALK *fusion status with results characterizing the presence of *EGFR *and *KRAS *mutations in the same set of cancer samples revealed that *ALK *fusions occurred in the absence of *KRAS *mutations. Previous reports have indicated that *ALK *fusion can occur concurrently with *EGFR *mutations (1/305) and *KRAS *mutations (1/120), although these may be rare events [30,33]. Consistent with these observations, we also identified a patient with concurrent *EGFR *exon 19 deletion and *EML4*-*ALK *fusion. The patient, who was female, non-smoking, and Chinese with histological adenocarcinoma, exhibited post-surgery overall survival of more than 38 months. This observation was particularly interesting because of the abnormality of mutations in two potential "tumor-driving" receptor genes in a single tumor. Whether tumor progression is dependent on both kinases, or only one of the two receptors, is unclear in this case. Thus, clarification of the actual activation status of the two receptors is important in assessing the use of targeted therapies, including EGFR inhibitors and/or ALK inhibitors. Notably, the frequency of *ALK *fusions in patients with adenocarcinomas that did not exhibit *EGFR*/*KRAS *mutations reached 45.0% (9/21), strongly indicating that only a specific molecular subset of adenocarcinomas is characterized by *EML4*-*ALK *fusion. Thus, stratification of patients with *ALK *fusions may be useful in the development of personalized medical treatments for NSCLC.

Characterization of associations between *ALK *fusion status and clinicopathological variables revealed that *EML4*-*ALK *fusions were associated with adenocarcinoma, because fusions were identified in nine cases of adenocarcinoma, two cases of squamous carcinoma, and one case of adenocarcinoma with partial squamous carcinoma components. *EML4*-*ALK *fusion was also associated with non-smokers (*P *= 0.02), and patients who possessed the *ALK *fusion had significantly less smoking pack-years (6.9 vs. 19.1; *P *= 0.01). Grouping of patients revealed that the frequency of the *EML4*-*ALK *fusion was 16.13% (10/62) in patients with adenocarcinomas and 19.23% (10/52) in never-smokers. Patients with *EML4*-*ALK *fusions were also significantly younger (53 vs. 61; *P *= 0.03) in comparison to patients without the fusion gene. Results showing that *ALK *fusion is frequently associated with adenocarcinoma and younger onset of disease were consistent with previous reports [37].

No mutation in *ALK *was identified by sequencing samples from 50 NSCLC cases (data not shown). However, patients in the *EML4*-*ALK *cohort appeared to exhibit increased survival, compared to patients without the fusion, although this was not statistically significant (*P *= 0.15), with a hazard ratio of 0.54 (95% CI, 0.23-1.26) for overall survival. Considering that mutated *EGFR *is used as a prognostic indicator, better prognosis may be associated with the presence of *EML4*-*ALK *fusions in NSCLC, suggesting that *EML4*-*ALK *may represent an effective prognostic factor, similar to *EGFR*.

Recently, *ALK *fusion was reported to play a role in resistance to EGFR tyrosine kinase inhibitors (TKIs) [34]. However, cancers characterized by expression of *ALK *fusions are clinically-sensitive to specific ALK inhibitors [25,26]. Thus, *ALK *fusions may be useful as biomarkers for predicting the response to ALK TKI and resistance to EGFR TKI. Immunohistochemical analysis of ALK expression could serve as a screening tool and potential surrogate technique to assess *ALK *fusion status. Though the correlation between *ALK *gene rearrangement and EML4-ALK mRNA and protein levels has been disputed [33], [38], [39], our data suggests that *ALK *fusion did correlate with increased mRNA expression.

In the present study, patients with *ALK *fusions did not receive TKI treatment. However, our results, together with those from previous studies, indicate that correlations between *ALK *fusion status and the efficacy of EGFR TKI and ALK TKI require further investigation in a larger patient cohort. Concurrent *EGFR *mutation and *EML4*-*ALK *fusion in one patient in this study (1/12), together with data from previous reports [30,33], also suggests that treatment efficacy should also be assessed in this group of patients. Given the reported resistance of tumors with *ALK *fusions to EGFR TKI, in rare cases with concurrent *ALK *fusion and *EGFR*/*KRAS *mutation, the molecular heterogeneity of these altered oncogenes and potential cross-talk between their protein products requires further investigation. Results from such studies would be useful in the development of sequential or concurrent target therapies.

In summary, RACE-coupled PCR sequencing can serve as sensitive tool to identify *ALK *fusions with a variety of potential partners in patient samples. However, in the present study of NSCLC samples, only *EML4 *was identified as a partner protein. A high frequency (11.6%) of NSCLC samples exhibited expression of different *EML4*-*ALK *transcript variants. Patients with *ALK *fusions appeared to exhibit a trend towards improved survival in comparison to patients without fusions. Notably, the presence of ALK fusions was associated with histological adenocarcinomas and with wild-type *EGFR *and *KRAS *status, which may be of clinical relevance in targeted therapy using ALK inhibitors.

## Conclusions

A high frequency of the *EML4*-*ALK *fusion gene was present in Chinese patients with NSCLC, particularly in patients with adenocarcinomas without *EGFR*/*KRAS *mutations. *ALK *fusion status was significantly associated with ALK mRNA expression levels. RACE-coupled PCR sequencing was highly sensitive and could serve as a method for identifying *EML4*-*ALK *fusion patients for targeted therapies in the clinic.

## Methods

### Lung cancer patients

In total, 103 NSCLC specimens were obtained from patients who underwent surgery for NSCLC from 2004 to 2006 at Guangdong General Hospital in the Guangdong Lung Cancer Institute of the Guangdong Academy of Medical Sciences. NSCLC samples included 62 adenocarcinomas, 29 squamous cell carcinomas, 11 large cell carcinomas, and 1 smooth muscle sarcoma.

Informed consent to use resected tissue for genetic analysis was obtained for all patients. The study was approved by the Institutional Review Boards (IRBs) of Guangdong General Hospital.

Demographic and clinicopathological profiles for the cases are shown in Table 3. Staging and histological classification were based on the World Health Organization (WHO) system. All 103 cases were analyzed for *ALK *fusion status, and both *EGFR*/*KRAS *mutation data and *ALK *fusion data were obtained for 96 of the 103 cases.

**Table 3:** Summary of patient demographic and clinicopathological profiles.

Characteristic	Number of Patients	ALK fusion		
		
		Positive	Negative	*P*-value
No. of patients	103	12 (11.6%)	91	
Age (years; mean ± SD)	52 (< 61 y)	8	44	0.37
	53 (≥ 61 y)	4	39	
Gender				
Male	74 (71.8%)	7	67	0.27
Female	29 (28.2%)	5	24	
Smoking				
Smokers	51 (49.5%)	2	47	0.03
Non-smokers	52 (50.5%)	10	44	
Histology				
Adenocarcinoma	62 (60.2%)	10	52	0.08*
Squamous cell	29 (28.1%)	2	27	
carcinoma	11 (10.7%)	0	11	
Large-cell	1 (1.0%)	0	1	
carcinoma				
Others				
Stage				
I	63 (61.2%)	8	55	0.72
II	18 (17.5%)	1	17	
III	20 (19.4%)	3	17	
IV	2 (1.9%)	0	2	

* AC compared with combined SCC, LCC, and other histological types.

### Nucleic acid extraction

Total RNA and DNA were extracted from lung tissue samples using the RNeasy kit (QIAGEN, Valencia, CA) and DNeasy kit (QIAGEN), respectively. All samples were pathologically assessed and trimmed at low temperature to ensure that more than 80% of the sample consisted of tumor tissue, while protecting the RNA from degradation. RNA quality was assessed by gel electrophoresis using an Agilent bio-analyzer 2100 (with RNA integrity number [RIN] > 6). DNA quality was assessed by routine gel electrophoresis.

### *ALK *fusion analysis by RACE-coupled PCR sequencing

This methodology is based on the principles of 5'-RACE and RT-PCR. Briefly, an *ALK *gene-specific primer was used to reverse transcribe RNAs into cDNAs. The sequence of the primer was 5'-TTCAGGCAGCGTGTTCACAGCCA-3'. Reverse transcription was performed based on the manufacturer's recommended protocol for the TaKaRa RNA PCR (AMV) kit, Ver. 3.0 (Takara). Briefly, the RT reaction consisted of 2 μL MgCl_2_, 1 μL 10 × RT buffer, 3.75 μL H_2_O, 1 μL 10 mM dNTPs, 0.25 μL RNase inhibitor, 0.5 μL AMV, 0.5 μL 12.5 μM gene-specific primer, and 1 μL RNA (< 500 ng total RNA). The reaction was incubated at 42°C for 30 min, 99°C for 5 min, and 5°C for 5 min. After reverse transcription, cDNAs were purified using the High Pure PCR Product Purification Kit (Roche) according to the manufacturer's protocol. Purified cDNAs were further subjected to poly-cytidine (poly-C) tailing. Briefly, reactions containing 1.5 μL 1% BSA, 5.0 μL 5 × tailing buffer, 2.5 μL 2 mM dCTP, and 15.0 μL purified cDNA were gently mixed and incubated for 2-3 min at 94°C, followed by incubation for 2 min on ice. After the addition of 1 μL TdT, the reactions were incubated for an additional 30 min at 37°C. TdT was then inactivated by incubation at 65°C for 10 min, and the contents of the reaction were collected by brief centrifugation and placed on ice.

Two PCR reactions were performed to amplify target cDNA fragments spanning exon 20 of *ALK *and upstream sequences that may contain transcript sequences for any genes fused to *ALK*. Primers for the first and PCR reactions were as follows: forward primer 5'-GGCCA CGCGTCCACTAGTACGGGGGGGGGG-3' and reverse primer 5'-GGCACCTCCTTCAC GTCACTGATG-3' and forward primer 5'-GGCCAC GCGTCGA CTAGTAC-3' and reverse primer 5'-ACCAGGAAACAGCTAT GACCGGTCTTG CCAGCAAAGCAGTAG TTG -3', respectively. PCR reactions were performed according to the manufacturer's instructions for the HS PCR kit (Takara). PCR products were then purified and labelled using the BDT v3.1 Ready Reaction-5000 and SEQ Buffer 5000 Ready Reaction (Applied Biosystems) using the M13 sequencing primer, 5'-CAGGAAACAGCT ATGACC-3'. Sequencing was performed on a Genetic Analyzer 3730XL (Applied Biosystems). Target sequences of interest were aligned with the *ALK *reference sequence (NM_004304.3) to determine if a fusion with another gene was present.

### EGFR mutation analysis by direct sequencing

Genomic DNA from each sample was used for sequence analysis of *EGFR *exons 18, 19, 20, and 21. These exons were amplified by PCR as previously reported [40], and the resulting PCR products were purified and labelled for sequencing according to the manufacturer's protocol for the BigDye 3.1 kit (Applied Biosystems).

### Statistical analyses

Statistical analysis of associations between *ALK *fusion status and clinical factors was performed using the chi-squared test or Fisher's exact test. Student's *t*-test was used for comparisons between means. Pearson's correlation coefficient was calculated when assessing relationships between continuous variables. Kaplan-Meier survival curves were generated and the log-rank test was used to test the significance of differential survival. *P *values less than 0.05 were deemed to indicate statistical significance.

## Competing interests

X. Zhang is the vice president and head of the AstraZeneca Innovation Center China (ICC). He is a member of the AstraZeneca global senior management team for the cancer and infection research areas. M. Zhu and L. Yin are senior scientists, and S. Zhang is a basic scientist at the AstraZeneca Innovation Center China. A patent application has been submitted for the RACE-coupled PCR sequencing method for *ALK *fusion detection.

## Authors' contributions

XZ performed experiments and prepared the manuscript. SZ, LY, JA, ZX, JL, and SC performed experiments. XY, JY, and QZ helped to prepare tissue samples and obtained informed consent from the patients. XZ, MZ, and YW designed the experiments, supervised the project, and prepared the manuscript. All authors read and approved the final manuscript.

## Note

The English in this document has been checked by at least two professional editors, both native speakers of English. For a certificate, please see:

http://www.textcheck.com/certificate/TfEIdB
